# Activity of Phosvitin in Hydroxyapatite Acid-Damage Immersion and Antimicrobial Assays

**DOI:** 10.1155/2020/8831311

**Published:** 2020-10-24

**Authors:** Richard D. Shipman, Sean D. Doering, Jack R. Hemsath, Eun Joo Lee, Jennifer E. Grant

**Affiliations:** ^1^The Applied Science Program, University of Wisconsin-Stout, Menomonie, WI 54751, USA; ^2^The Applied Biochemistry and Molecular Biology Program, University of Wisconsin-Stout, Menomonie, WI 54751, USA; ^3^Food and Nutrition Department, University of Wisconsin-Stout, Menomonie, WI 54751, USA; ^4^Biology Department, University of Wisconsin-Stout, Menomonie, WI 54751, USA

## Abstract

Phosvitin, the most highly phosphorylated metal-binding protein found in nature, binds more than 100 calcium ions, and has been identified as an agent that could be used to generate biomineralization scaffolds. Because of published reports describing phosvitin's affinity for calcium and potential antibiotic activity, this study was undertaken in order to evaluate phosvitin for both antibiotic activity against common microorganisms and the ability to protect hydroxyapatite surfaces from acid damage. To more clearly define its antibiotic action, the effects of phosvitin on *Micrococcus luteus*, *P. mirabilis*, *B. cereus*, *E. coli*, and *S. epidermidis* were evaluated. In both Kirby–Bauer tests and liquid culture growth inhibition assays, phosvitin inhibited *M. luteus*, a microorganism that thrives in the human mouth, but not the other bacteria tested. The MIC of phosvitin was determined to be 31.3 *μ*g/mL when delivered in 1 mM CaCl_2_ but was 0.5 mg/mL in the absence of added calcium. Expanding on the potential impacts of phosvitin on the mouth, its action was evaluated in a model of tooth decay represented by acid-damaged hydroxyapatite discs. SEM, AFM, and FAAS analyses revealed that pretreatment of discs with phosvitin modulated the damage-induced morphology and topography changes associated with acid-damaged discs.

## 1. Introduction

Tooth decay affects 45.8% of the US population aging from 12 to 19 years old [1]. Prevention of tooth decay is a major goal, starting with education about diet and dental hygiene, but also encompassing potential future treatments that may involve the use of bioactive agents to either protect or augment dental enamel [2–5]. Agents that are being tested as enamel protective agents include hydroxyapatite nanoparticles [3, 4, 6] and calcium-binding peptides [5, 7]. At the same time, including an antibiotic in a dental treatment could benefit patients. Peptides and proteins can contribute towards antibiotic activity; in addition to reports that anionic peptides harbor antibiotic potential in general [8], several calcium-binding peptides have been identified as having antibiotic properties and may also provide a calcium reservoir [9, 10]. Considering that an agent that promotes both calcium deposition and antibiotic activity might promote oral health, properties of the hen egg yolk protein, including calcium-binding and anionic character, suggested that phosvitin provides a strong candidate for a tooth decay preventative.

Phosvitin is the most phosphorylated protein found in nature [11, 12] and binds up to 127 calcium ions at pH 6.5 [12]. Accounting for 60% of chicken egg yolk phosphoproteins, this protein possesses redox activity [13], facilitates biomineralization [14, 15], and acts as an antibiotic when bacteria are placed under stress [16, 17]. In addition to undergoing calcium-induced conformational changes [18–20], phosvitin is thought to form calcium–phosvitin aggregates in the presence of high concentrations of calcium and has hydrophobic patches at its N^−^ and C^−^termini [18, 19]. For several reasons including its general similarity to proteins associated with tooth mineralization, phosvitin would seem to present an outstanding candidate for use in protecting enamel. However, its interaction with hydroxyapatite surfaces has not been described, and data on its antimicrobial effects are limited.

Phosvitin antimicrobial activity was tested against a panel of bacteria that were chosen not only for their availability, but also because they are often part of the human biome—some in health and others in disease. *Proteus mirabilis* can often be found in the gastrointestinal system and can cause urinary tract infections [21, 22]. *Bacillus cereus* is a Gram-positive toxin-producing bacterium that is common to soils and in foods and can cause infections of the intestinal tract, the eye, and the lungs and of wounds [23, 24]. *Escherichia coli* is the most abundant aerobic bacterium residing in the human gut, and although several strains are a commensal part of the human microbiota, some strains cause infections [25, 26]. While the benign form is commensal and largely innocuous flora of the human skin [27], the invasive form of *Streptococcus epidermidis* is the leading cause of infections due to placement of surgical materials [28]. And finally, while *Micrococcus luteus,* a microorganism common to the human skin and mouth [29], is not known to pose any significant danger to healthy humans, it has been linked to pathology in compromised patients [30, 31]. *Micrococcus luteus* may also be a causal factor in formation of dental caries [32].

The research presented here indicates that phosvitin harbors antibiotic activity towards *Micrococcus luteus*, but not towards other bacteria tested in Kirby–Bauer disc diffusion assays. At the same time, the presence of phosvitin in a calcium phosphate precipitation assay revealed that inclusion of phosvitin increased the amount of precipitate formed. In addition, SEM, AFM, and FAAS analyses of measures of damage to hydroxyapatite discs demonstrated that treatment with phosvitin modulates acid-induced damage to hydroxyapatite.

## 2. Materials and Methods

Hydroxyapatite discs were purchased from Clarkson Chromatography Products. Phosvitin was a kind gift from Dr. Dong Ahn at Iowa State University and was prepared according to Lee's production method [33–35]. Additional materials were acquired from Thermo Fisher Scientific unless otherwise noted.

### 2.1. Alizarin Red Hydroxyapatite Crystal Assay

The Alizarin Red Hydroxyapatite Crystal Assay was based on Microtiter Plate Hydroxyapatite Inhibition Assay by Xiao et al. (2015) [36]; however, modifications were made that allowed the assessment of calcium phosphate precipitation and compensated for the presence of a highly acidic environment. Phosvitin and phosvitin-derived peptide digest solutions were made in duplicate in 50 mM HEPES buffer solution at pH 7.4 at fixed concentration of 10 *µ*M. To a new 1.5 mL Eppendorf tube, 500 *µ*L of each solution was added then mixed with 10 *µ*L of both 1.0 M calcium chloride and 0.6 M diammonium phosphate to form hydroxyapatite precipitation in solution. The mixtures were incubated at room temperature for 4 hours to allow for precipitation. Sample were then centrifuged at 10,000 rpm for one minute to form a pellet, whereupon excess supernatant was carefully removed. The pellet was then stained with 500 *µ*L of 5% alizarin red solution in 75% ethanol. Twice, the slurry was vortexed for 30 seconds and centrifuged at 10,000 rpm; the pellet was then washed with a 75% ethanol wash solution to remove any remaining free alizarin red stain. To dissolve the hydroxyapatite, the final resuspended pellet was placed in 30% acetic acid with 100 *µ*L mineral oil to prevent evaporation and heated on a thermal mixer at 85°C for 15 minutes at 300 rpm. Then, 200 *µ*L of each sample was transferred to a 96-well microtiter plate and the absorbance at 405 nm was determined. The percent change in hydroxyapatite formation for a given phosvitin calculation was determined by taking the ratio of the measurement from the phosvitin experiment to that of the control sample that contained no phosvitin. A standard curve was produced by mixing appropriate volumes of calcium and phosphate solutions at a 1:1 ratio in a 50 mM HEPES buffer solution.

### 2.2. Hydroxyapatite Disc Acid-Damage Immersion Assays

The acid-immersion model of tooth decay developed by Lim et al. (2009) [37] was used to measure the effects of phosvitin when incubated with the discs. A hydroxyapatite disc was placed in 5 mL of reverse osmosis (RO) water in a 15 mL falcon tube and was adjusted to pH 3.26 with hydrochloric acid and then treated with a phosvitin solution ranging from 0.125 to 1.0 mg/mL and allowed to incubate for one month. Then, discs were removed from the acidic solutions and dried overnight by vacuum centrifugation. All experiments were performed in triplicate. SEM analysis was performed to assess disc surface morphological changes, while AFM measured disc surface topographical changes. The extent to which calcium was released from discs was determined by FAAS of the immersion solution at the conclusion of the experiment.

### 2.3. Scanning Electron Microscopy

Dried hydroxyapatite discs were fixed to a scanning electron microscopy mount with carbon tape. The disc mounts were then placed in a Cressington Sputter Coater 108 Auto and coated with a layer of gold particles for one minute at full voltage. SEM images were acquired with a TESCAN Vega 2 Scanning Electron Microscope at a 20.0 working distance using a secondary electron detector at an accelerating voltage of 20 kV. Imaging of each sample was performed in triplicate; the specific details associated with the recorded images are presented in the figure legends.

### 2.4. Atomic Force Microscopy

Acid-treated discs were mounted to AFM metal mounts with carbon tape then placed onto the main stage area of a Bruker Innova Atomic Force Microscope. Each disc was aligned with the AFM laser and cantilever; acquisition settings are reported with the corresponding figure.

### 2.5. Flame Atomic Absorption Spectroscopy

Our protocol largely followed that provided by the Environmental Protection Agency [38] and as reported in the published literature [39, 40]. To release protein-bound calcium into solution, samples were diluted 1:20 into ddH_2_O and placed in a 50 mL cylindrical reflux tube, to which 0.4 mL of 1:1 nitric acid and 0.2 mL 1:1 hydrochloric acid was added. The immersion solution was then heated at 100–105°C using a heat block; sample volume was reduced to 10 mL and refluxed at 95°C for 30 minutes. Cooled samples were diluted to 20 mL with ddH_2_O then passed through a 0.2 *µ*m filter to remove particulates.

Just prior to analysis, nitric acid-digested samples (20 mL) were treated with 100 *µ*L of lanthanum solution to depress the response from oxyanions such as free phosphates, aluminates, and silicates that are present after the digestion. Calcium concentration was determined against a calcium stock standard set in serial dilution of 1 to 3 ppm. All measurements were performed in triplicate.

Flame atomic absorption spectroscopy was performed using a Thermo Elemental Solaar S-Series Atomic Absorption Spectrometer operated under acetylene gas (10 psi) mixed with air (30 psi). Additional instrument settings are provided in the relevant figure legends.

### 2.6. Kirby–Bauer Tests

The antimicrobial activity of phosvitin towards *M. luteus*, *P. mirabilis, S. marcescens, P. aeruginosa, B. cereus*, *E. coli*, and *S. epidermidis* was examined using Kirby–Bauer disc diffusion assays [41]. Solutions of 25 mg/ml phosvitin, 25 mg/ml phosvitin in 1 mM ZnCl_2_, and 25 mg/ml phosvitin in 1 mM CaCl^−^_2_ were prepared directly from a lyophilized powder. Bacteria were grown in Luria–Bertani (LB) broth at 37°C for 24 hours in shakers at 250 rpm; then a lawn of bacterial solution was spread across the plate. Plates were treated with discs created from autoclaved filter paper that was dipped into the proper phosvitin solution immediately before placement. Lawns were cultivated at 37°C for 48 hours to obtain the zones of inhibition. Discs treated with 1 mM CaCl_2_ and 1 mM ZnCl_2_ solutions were used as controls.

### 2.7. Minimum Inhibitory Concentration Determinations

The minimum inhibitory concentration (MIC) of phosvitin towards *M. luteus* and *B. cereus* was determined according to the method of Andrews (2001) [42]. These MIC determinations were made using phosvitin that was dissolved in 1 mM CaCl_2_ to ensure maximal loading of calcium on the protein. A twofold serial dilution was prepared using concentrations of calcium chloride bound phosvitin from 1 mg/ml to 3.9 *μ*g/ml in LB broth with 1 mM CaCl_2_ LB broth as a control. Bacteria were grown in LB broth at 37°C for 24 hours and 10 *μ*L of bacterial solution was used to inoculate dilutions. Treatments were cultivated at 37°C for 48 hours at 250 rpm on a shaker.

The ability of phosvitin to impair the growth of colonies of *M. luteus* in solution in the absence of added calcium was also determined, as were the effects of casein, or bovine serum albumin (BSA). To achieve quantitation, turbidity was measured at an absorbance of 600 nm according to the method of Koch (1970) [43] using a Molecular Devices SpectraMax Microplate Reader.

## 3. Results

### 3.1. Measurement of Hydroxyapatite Formation

**Figure 1:**
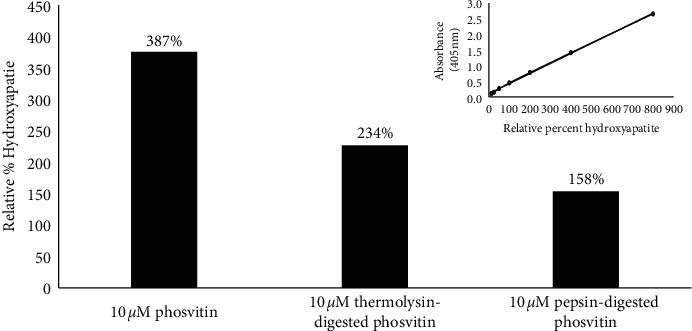
Extent of formation of calcium phosphate pellets in the presence of phosvitin. A calcium phosphate pellet assay was used to determine the effect of phosvitin or phosvitin digests on the extent of pellet formation. The amount of calcium retained in the pellet was determined using the alizarin red assay.

To measure the formation of hydroxyapatite, the method of Xiao et al. (2015) [36] was used. However, in order to accommodate the acidic solution of the acid damage assay, the method was modified. In addition to the changes to the wash strategy, the absorbance wavelength that was monitored was changed from 560 nm to 405 nm to accommodate a shift in the absorption profile of alizarin red in the acidic environment. Because of the shift in the absorption profile, the reported molar absorptivity of alizarin red at 560 nm could not be used to quantify the extent of hydroxyapatite formed. Instead, the percentage of hydroxyapatite formed in the presence of phosvitin was compared to the percentage formed in a control lacking phosvitin. As indicated in Figure 1, incubation of calcium phosphate in the presence of phosvitin increases the amount of hydroxyapatite that is formed. Samples incubated with phosvitin and phosvitin-derived peptides demonstrate an increase in the relative percent of hydroxyapatite formation. While full-length phosvitin showed a 387% increase in calcium pellet formation, thermolysin digests of phosvitin increased calcium pellet formation to 234%: however, pepsin-digested phosvitin only increased pellet formation to 158%. These preliminary studies suggest that crude digests of phosvitin are less effective than full-length phosvitin.

In general, the alizarin red assay provides a measurement of the extent of calcium phosphate formation as a pellet. However, this assay has weaknesses; subtle changes in pH cause differences in the spectral properties of the dye, and the calcium phosphate precipitate formed does not represent the hydroxyapatite found in enamel. To develop a more reliable assessment, an acid-damage model using hydroxyapatite discs was used; readouts included structural methods including SEM, AFM, and FAAS.

### 3.2. Scanning Electron Microscopy

**Figure 2:**
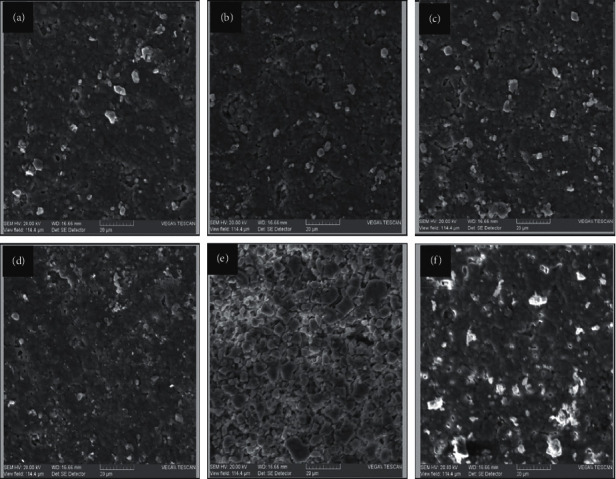
SEM micrographs of hydroxyapatite discs treated with phosvitin. Hydroxyapatite discs were damaged in the acid damage immersion assay in the presence of varying concentrations of phosvitin. Surface morphology was imaged with SEM at a working distance of 16.66 mm. (a) 1 mg/mL phosvitin. (b) 0.5 mg/mL phosvitin. (c) 0.25 mg/mL phosvitin. (d) 0.12 mg/mL phosvitin. (e) No phosvitin. (f) A hydroxyapatite disc not subjected to the acid damage immersion assay. The view field for each acquisition was 144.4 *μ*m wide.

Purchased hydroxyapatite discs were damaged in an acid immersion assay, and the influence of phosvitin pretreatment was assessed. The surface of the untreated acid-damaged discs was rough and contained pits and cracks (Figure 2(e)). Phosvitin-treated discs presented with smaller cracks; this was true of all concentrations of phosvitin treatments examined ranging from 0.125 to 1.0 mg/mL (Figures 2(a)–2(d)). Increased electron density was observed in small dots on the surface of phosvitin-treated discs, an indication of the formation of a layer of electron-rich protein, the phosvitin, on the surface of the hydroxyapatite. An untreated disc was analyzed for comparison (Figure 2(f)). The entire experiment was repeated in duplicate.

### 3.3. Atomic Force Microscopy

**Figure 3:**
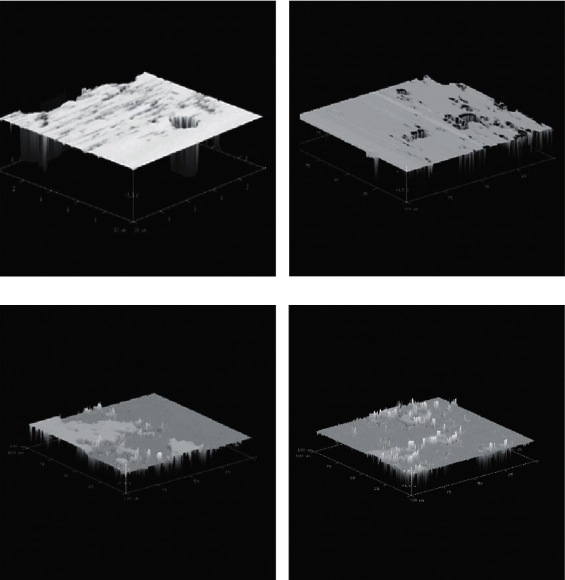
AFM captures of hydroxyapatite discs. (a) A disc straight out of the package. (b) An acid-damaged hydroxyapatite disc. (c) A disc treated with 1.0 mg/mL phosvitin and (d) a disc treated with 1 mg/mL BSA were exposed to acid damage for one month and then imaged by AFM.

**Figure 4:**
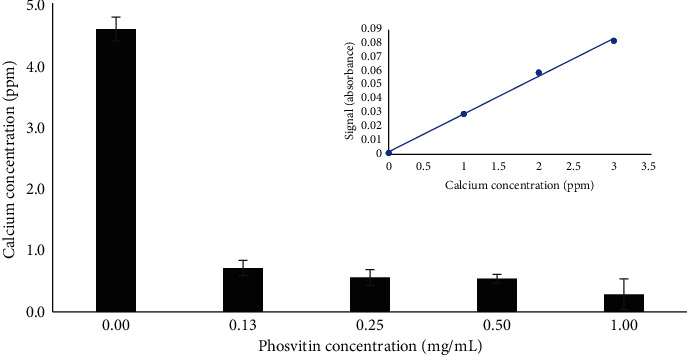
Effect of addition of phosvitin on calcium release form hydroxyapatite discs exposed to the month-long acid-damage immersion assay. Discs were treated with phosvitin solutions ranging from 0.13 to 1.0 mg/mL phosvitin. FAAS measurements of the calcium released into solution were taken at the initial onset of the experiment and also after thirty days. A correction for calcium contributed by the phosvitin solution was made by subtracting the initial time measurement from that after thirty days. Inset: standard curve relating calcium concentration to FAAS signal; error measurements were calculated but were inside the span of the markers.

The surface topography of the acid-damaged hydroxyapatite discs was assessed using AFM (Figure 3). Untreated (Figure 3(a)) and acid-damaged hydroxyapatite discs (Figure 3(b)) possessed a rough surface, corresponding to acid damage that reduces the calcium content of the discs. Acid-damaged discs that had been incubated in the presence of phosvitin solution, however, exhibited a smoother AFM profile presumably indicating less acid damage. Indeed, there were areas of raised surface that could potentially indicate thicker areas of phosvitin deposition (Figure 3(c)). To understand whether a globular protein could prevent the development of surface roughness, the acid-damage immersion assay was performed in the presence of 1 mg/mL bovine serum albumin (Figure 4(c)). While the BSA-treated discs presented an uneven surface topography, the AFM profile of these discs did not fully recapitulate the extent of pit and crack formation associated with protein-naïve hydroxyapatite discs.

### 3.4. Flame Atomic Absorption Spectroscopy

The solution that was retained after a hydroxyapatite immersion experiment was subjected to flame atomic absorption spectroscopy (FAAS) in order to quantify the extent of release of calcium ions in solution.

Figure 4 illustrates how treatment of a hydroxyapatite disc with increasing concentrations of phosvitin affects the amount of calcium released from discs after for one month of immersion at pH 3.26. The inset in Figure 4 presents the standard curve that was used to calculate calcium levels in experimental samples from zero to three ppm to FAAS signal; linear regression yielded an equation for the line that possessed an *R*^2^ of 0.9972. Phosvitin-naïve discs immersed in a pH 3.26 solution for one day released an average of 0.557 ppm calcium ions (*n* = 6) in solution, whereas after one-month time the data report an average of 4.6 ± 0.2 ppm calcium ions released into solution. However, increasing concentrations of phosvitin up to 1 mg/mL resulted in diminished calcium release in comparison to phosvitin-naïve discs. The concentration of calcium ions released was 0.7 ± 0.1 ppm, 0.5 ± 0.1 ppm, 0.54 ± 0.07 ppm, and 0.3 ± 0.2 ppm when discs were treated with phosvitin at concentrations of 0.13 mg/mL, 0.25 mg/mL, 0.5 mg/mL, and 1 mg/mL, respectively. Regardless of the concentration of phosvitin tested, incubation of discs with the protein resulted in significant retention of calcium ions in this acid damage assay.

Taken together, the SEM, AFM, and FAAS results demonstrate that phosvitin is mitigating damage to hydroxyapatite discs in the acid immersion experiments. SEM data provides a qualitative description of how treatment of discs with phosvitin leads to surface morphology changes that reflect a reduction in cracks compared to damaged discs naïve of phosvitin. The AFM data indicates an overall decrease in surface roughness. The third and final set of data to be taken into consideration is the FAAS data that quantified the extent to which phosvitin impeded the dissolution of calcium ions from hydroxyapatite discs at all concentrations tested.

### 3.5. Kirby–Bauer Tests

**Figure 5:**
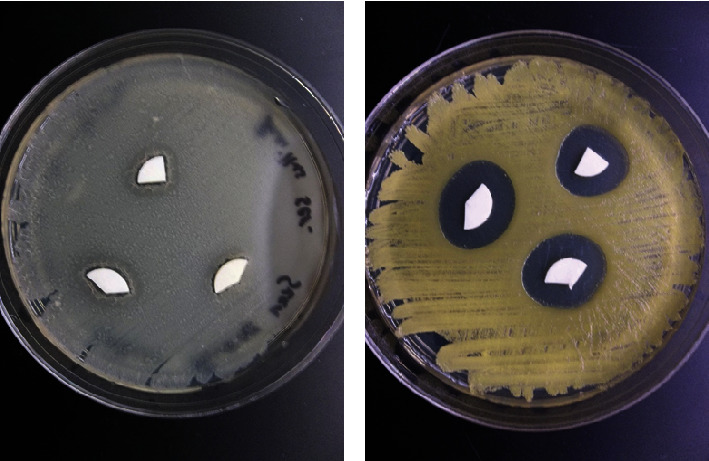
Kirby–Bauer disk diffusion assay. (a) *Bacillus cereus.* (b) *Micrococcus luteus*. Both bacteria were grown on LB agar. Disks were treated with a 25 mg/ml phosvitin solution containing 1 mM ZnCl_2_.

Kirby–Bauer test showed inhibition of growth around treated discs in some bacterial species. *B. cereus* was slightly inhibited by 25 mg/ml phosvitin in 1 ZnCl_2_ and 25 mg/ml phosvitin in 1 mM CaCl_2_ (Figure 5(a)). *M. luteus* was inhibited by all discs treated with phosvitin ranging from 1 to 25 mg/ml and with either 1 mM CaCl_2_ ((Figure 5(b)) or 1 mM ZnCl_2_; no other bacteria were inhibited by disc treatments at any concentration.

### 3.6. Minimum Inhibitory Concentration Experiments

To further explore the data from the Kirby–Bauer tests, the antimicrobial inhibition of *B. cereus* and *M. luteus* was determined visually using the MIC serial dilution strategy. Both phosvitin and 1 mM CaCl_2_ were included in the assay. In these experiments, *B. cereus* did not have visible inhibition at any concentration less than 1 mg/ml treatment, while the MIC of *M. luteus* was determined to be slightly greater than 31.3 mg/mL.

**Figure 6:**
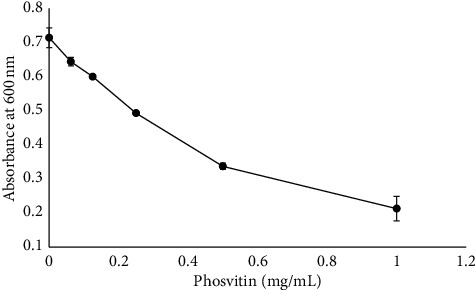
Minimum inhibitory concentration assay. The ability of phosvitin to inhibit the growth of *M. luteus* was assessed in the absence of added calcium by measuring turbidity at an absorbance of 600 nm. Error bars reflect the standard deviation associated with the average of two measurements. There was no visible growth of *M. luteus* at either 0.5 mg/mL phosvitin or at 1 mg/mL phosvitin. When linear regression analysis was performed on the data at 0.13, 0.25, and 0.5 mg/mL phosvitin (trend line not shown), the *R*^2^ for the linear regression was 0.9906, and the equation for the line was *y* = −0.7408*x* + 0.6969.

1 mM CaCl2 represents a strong concentration of calcium ions. Whether the inclusion of 1 mM calcium ions was affecting *M. luteus* caused concern, and the authors were also concerned that inclusion of 1 mM CaCl_2_ in any potential pharmaceutics could prove deleterious to human health. Therefore, the phosvitin-dependent inhibition of the growth of *M. luteus* was also evaluated in the absence of added calcium. And while inhibition of bacterial growth was observed in the absence of added calcium, the MIC was increased to 0.5 mg/mL (Figure 6). Neither casein, a phosphorylated protein known to participate in amorphous calcium phosphate nanocomplexes [44], nor bovine serum albumin inhibited *M. luteus* to any appreciable extent (unpublished results). Overall, these data indicate that phosvitin robustly inhibited *M. luteus* when provided in the presence of calcium, but only modestly in the absence of added calcium. Use of bovine serum albumin nor casein reproduced these effects.

### 3.7. Application of Findings in the Context of the Literature

There is a prior report of phosvitin proving bactericidal towards *E. coli* and *S. aureus* in a model of infection of zebrafish embryos [45], indicating that phosvitin exhibited antimicrobial action towards bacteria placed under stress. For the first time, this study revealed antimicrobial action of phosvitin towards *M. luteus*, a common Gram-positive to Gram-variable microorganism of the human microflora [46], expanding the role of phosvitin as an antimicrobial agent acting against *M. luteus*. While *M. luteus* is not considered the main causative agent in dental caries [47], it is associated with them [48, 49]. An opportunistic pathogen, it is considered largely unharmful, except for isolated reports associated with human cases of septic arthritis [50], septic arthritis [51], meningitis [52], and pneumonia [53].

Our data indicates that full-length phosvitin is potent as an antibiotic agent towards *M. luteus* when delivered with 1 mM CaCl_2_, having an MIC of 33 mg/mL, a rather potent value. However, this potency is lost in the absence of calcium; furthermore, phosvitin did not inhibit other microorganisms tested to an appreciable extent. The relevance of calcium in phosvitin has been reported in the literature; phosvitin's three-dimensional conformation at air–water interfaces is dependent on calcium [18]. Indeed, while phosvitin may occupy a more compact conformation at low calcium levels, at high calcium concentrations, Belhomme et al. (2007) [18] propose that phosvitin adopts an extended brush conformation that promotes the formation of calcium/phosvitin protein aggregates. It is tempting to speculate that the brush conformation may participate in antibacterial activity towards *M. luteus*.

Phosvitin increased calcium formation in a calcium phosphate precipitation assay and mitigated acid damage to hydroxyapatite discs. This is consistent with the data from Belhomme et al.[18] which suggested formation of extensive calcium/phosphate aggregates. Coupled with the antibiotic data presented in this report, it would seem that phosvitin presents an ideal candidate for inclusion in formulations that strengthen teeth. The fact that phosvitin can participate in micelles [54] suggests that it may lend itself to formulations convenient and effect in dental and antibiotic applications, from the standpoint of both interaction with hydroxyapatite surfaces and drug delivery.

There is also some interest in whether component peptides of phosvitin can bind calcium and/or retain biological activity. While we examined whether thermolysin or pepsin digests of phosvitin demonstrated activity in our assays, the unpurified digests did not stimulate formation of a calcium pellet to the same extent as full-length phosvitin (Figure 1); neither did they exhibit meaningful antibiotic activity (unpublished results). This can potentially be addressed by further concentrating the peptide digests or even partially purifying component fractions to the point, future studies that may lead to the identification of bioactive peptides.

## 4. Conclusion

The SEM, AFM, and FAAS analyses reported here indicate that treatment of hydroxyapatite discs with phosvitin protect the discs against acid damage. Furthermore, phosvitin has been shown to possess antimicrobial activity towards *M. luteus*, but not against other bacteria tested. For these reasons, phosvitin may prove to be of great interest in maintaining the health of the mouth both in terms of protecting hydroxyapatite of teeth against acid damage and against potential cariogenic activity *M. luteus,* a microorganism common to the mouth.

## Data Availability

The data used to support the findings of the study are available from the corresponding author upon request.
